# Udder Hygiene and Mastitis Indicators in Contrasting Environmental Conditions during Half-Time Confinement in Pasture-Based Dairy Systems

**DOI:** 10.3390/ani13091544

**Published:** 2023-05-04

**Authors:** Graciana R. Mendina, Juan Pablo Damián, Ana Meikle, Pablo Chilibroste, Oscar Bentancur, Maria de Lourdes Adrien

**Affiliations:** 1 Facultad de Veterinaria, Universidad de la República, Ruta 3 km 363, Paysandú 60000, Uruguay; 2 Facultad de Veterinaria, Universidad de la República, Ruta 8 km 18, Montevideo 13000, Uruguay; 3 Facultad de Agronomía, Universidad de la República, Ruta 3 km 363, Paysandú 60000, Uruguay

**Keywords:** compost barn, outdoor soil-bedded, mud, rainfall, moisture

## Abstract

**Simple Summary:**

Current intensification in pasture-based systems demands periods of part-time confinement or even complete confinement when pastures are limited. Outdoor soil-bedded yards are commonly used in these systems but can predispose cows to dirtier udders and, therefore, a greater risk of mastitis. The aim of the study was to compare the effect of two types of housing, compost barn and outdoor soil-bedded yard, on udder hygiene and mastitis indicators in Holstein dairy cows calved in autumn and spring. In both treatments, the cows were confined during one interval between milking sessions when a supplement was offered and went out to graze in the other interval. The outdoor soil-bedded group presented a greater percentage of dirty cows compared to the compost barn in both calving seasons. Additionally, outdoor soil-bedded autumn calving cows were dirtier after rain than on days without previous rain. However, no differences in somatic cell count or prevalence of mastitis were found between cows in outdoor soil-bedded and compost barn confinement.

**Abstract:**

This study aimed to compare the association between two types of housing, compost barns (CB) vs. outdoor soil-bedded yard (OD), and udder hygiene and mastitis indicators in Holstein dairy cows calving in autumn (*n* = 31) and spring (*n* = 27). After calving, cows were transferred to a pasture-based system with half-time confinement in one of two treatments: CB or OD. The udder hygiene score (UHS) was evaluated monthly and on days after rainfall over the entire lactation period. Individual somatic cell count (SCC) was determined throughout lactation, and the prevalence of intramammary infection (IMI) was estimated. Cows confined in OD presented higher UHS compared to cows in CB (*p* < 0.05) in both calving seasons. After rains, autumn-calving cows in OD were dirtier than on days without previous rain (OR = 1.85, CI 95%: 1.1–3.1; *p* < 0.02). However, no differences in IMI and clinical mastitis were found between OD and CB cows in either calving season.

## 1. Introduction

Although grazing systems have been associated with better indicators of animal health than confined systems [1,2], current intensification in pasture-based systems demands periods of part-time confinement or even complete confinement when pastures are limited [3]. Housing facilities are quite diverse, but outdoor soil-bedded yards (i.e., a soil-based yard with feeders with or without natural or artificial shade) are frequently used in South American pasture-based systems. Infrastructure and management in this type of confinement are simple and low-cost [4,5], but animals are exposed to environmental conditions such as rain, mud, or heat stress [6,7], and, therefore, animal welfare and productive sustainability could be at risk [3].

Several studies relate the environment of the cows with the degree of dirtiness of the animals [7,8,9]. The dirtiness score is an animal welfare indicator [10] and has been associated with udder health indicators. Schreiner and Ruegg [11] and Reneau et al. [12] found an association between the udder hygiene score (UHS) and intramammary infection (IMI) and somatic cell score (SCS) in dairy cows housed in free-stalls. The floor of outdoor soil-bedded systems under high moisture and manure accumulation may be associated with a high bacterial load that could predispose the cows to mastitis [13]. Cows are lying down 50 to 60% of the day with their teats in contact with the ground, thus exposing them to the bacterial load of the environment [13]. The compost barn (CB) emerged as a friendlier alternative from an animal welfare point of view, with positive impacts on udder health when compared to traditional free-stall systems [14], as the soft compost bedding pack can reach temperatures that inactivate mastitis-causing pathogens [14,15].

In addition, the environmental and climatic factors to which the animals are exposed may affect the UHS, for example, when there is a high incidence of rainfall and mud [16]. Depending on the calving season, animals are exposed to different conditions throughout their lactation, and indicators of udder health have been related to the environment and season [17,18,19]. As far as we are aware, only a few studies performed under complete confinement have evaluated the degree of cow cleanliness in outdoor systems when compared to indoor barns: Boyle et al. [20] and O’Driscoll et al. [7] reported higher dirtiness scores in non-lactating dairy cows on wood chip pads when compared to free-stall barns, whereas Sjostrom et al. [9] found lower UHS and incidences of clinical mastitis in lactating cows on outdoor straw packs when compared with a compost barn.

We are unaware of controlled studies evaluating the effect of different housing facilities in combination with grazing access, and with different climatic challenges throughout the entire lactation, on udder hygiene and health of dairy cows. We hypothesised that lactating dairy cows in outdoor soil-bedded yard, with greater exposure to the environment and adverse weather conditions, would have higher udder hygiene scores and, consequently, a higher prevalence of intramammary infection than cows in a compost-bedded pack barn. The present work aimed to compare the effect of the type of confinement (compost barn vs. outdoor soil-bedded yard) used in combination with grazing access on milk production, udder hygiene score, and somatic cell counts throughout the entire lactation in two calving seasons.

## 2. Materials and Methods

This study was performed at the Experimental Station, Dr. Mario A. Cassinoni, Facultad de Agronomía, Universidad de la República, Paysandú, Uruguay (32°23′07.6″ S 58°03′17.9″ W) between March 2019 and April 2020. Our study was part of a larger farmlet study designed to study the effect of environmental exposure on productive [21], economic, biological, and environmental parameters and the technological properties of milk [22].

### 2.1. Experimental Design

The study was performed with Holstein dairy cows that calved in March and April 2019 (*n* = 32; hereafter called autumn) and in July and August 2019 (*n* = 32; hereafter called spring). Before calving, cows were blocked according to the number of lactations, expected calving date, body weight (BW), and body condition score (BCS); all were healthy animals on clinical examination without a history of chronic mastitis. They were then randomly divided into two treatments: compost barn + grazing (CB) and outdoor soil-bedded yard + grazing (OD). The treatments started immediately after calving and lasted until the end of the corresponding lactation. All of the animals underwent a clinical examination between five and ten days postpartum to check their health status and ensure their continuity in the experiment. Due to calving complications or serious illnesses (caesarean section, anaplasmosis, traumatic reticulopericarditis, or mastitis at calving), some animals were removed from the experiment. Thus, the final number of animals was 31 in the autumn calving season (CB *n* = 16, OD *n* = 15) and 27 in the spring calving season (CB *n* = 13, OD *n* = 14).

The mean parity in autumn was 2.8 ± 1.4, BW at calving was 660 ± 78 kg, and BCS at calving was 3.0 ± 0.3. The mean calving date was 17 March 2019 ± 9.7 days, and the follow-up lasted until 6 February 2020. The mean parity in spring was 2.7 ± 1.2, BW at calving was 620 ± 64 kg, and BCS at calving was 2.8 ± 0.2. The mean calving date was 7 August 2019 ± 9.7 days, and the follow-up lasted until 4 May 2020.

Cows were confined during one milking interval, when a total mixed ration (TMR) was offered, and went out to graze in the other interval. In hot months (November to April), the grazing session took place between the afternoon and the morning milking to avoid heat stress, while in the remaining period, cows grazed between the morning and afternoon milking. Details of feeding components and management are reported in Méndez et al. [21]. Cows in CB were housed in a compost bedded-pack barn consisting of a roofed barn with ventilation (both natural and with fans) and sprinklers for cooling the animals. The area of the compost-bedded pack was 13.5 m^2^/cow. There was an adjacent concrete feeding area (6.75 m^2^/cow) with automatic water troughs and feed bunks with a linear space of 0.75 m/cow. The materials used in the bedded pack were eucalyptus, pine sawdust, wood chips, and rice husk stirred with a chisel plough twice a day. An 8 cm layer of new material was added every 15–20 days, depending on the temperature and moisture of the bedded pack. Cows in OD were confined in an outdoor soil-bedded yard. This yard had some initial grass cover but soon became poached. No bedding was added. This yard contained artificial shades of a wooden structure and plastic roof with a height of 4 m and an area of 4.8 m^2^/cow, automatic water troughs, and cement feed bunks with a linear space of 1.1 m/cow. The area was divided into two equal halves that were used alternately, depending on the accumulation of mud. The effective area of use was 72.8 m^2^/animal. Thus, grazing and housing periods as well as nutritional management in CB and OD cows were the same, except for the housing in which they stayed and received a TMR. It was necessary to completely confine the cows due to low pasture stock conditions from 11 to 21 April 2019, from 6 to 19 June 2019, and from 19 December 2019 to 1 January 2020. In these cases, animals were fed a TMR divided into two daily rations.

### 2.2. Routine and Measurements

All of the animals received the same pre-calving management. The cows entered the corresponding treatment after calving and were milked twice a day, at 04:00 h and 15:00 h in autumn-winter and 04:00 h and 16:00 h in spring-summer. Cows were forestripped to stimulate milk let-down and to detect clinical mastitis. They were then pre-dipped with a hydrogen peroxide-based sanitising solution (OxyCide^®^, GEA Farm Technologies, Inc., Naperville, IL, USA), dried with disposable paper towels, and after milking, teats were dipped in an iodine solution (LuxSan^®^ X, GEA Farm Technologies, Inc., Naperville, IL, USA).

Milk production was registered daily by an automatic recording system (GEA Farm Technologies, Inc.). Udder hygiene was scored during milking by the same person using a scale of 1 to 4: (1) completely free of or very little dirt, (2) slightly dirty, (3) mostly covered in dirt, or (4) completely covered by caked-on dirt [23]. The evaluations were carried out monthly and on days after rainfall higher than 30 mm. This cut-off point was considered the minimum amount of rain that would generate significant amounts of mud in our region, determined in the previous observations of our research group (unpublished data).

Individual milk samples were collected during both daily milkings to determine the SCC with a weekly frequency from calving to 90 days in milk (DIM), biweekly from 91 to 180 DIM, and monthly from 181 to 300 DIM. Samples were analysed by flow cytometry (Delta CombiScope™ FTIR 600 HP).

Somatic cell score (SCS) was calculated according to Shook et al. [24]: SCS = log2(SCC/100,000) + 3. To determine the monthly IMI, animals that had at least one instance of SCC higher than 200,000 cells/mL (or SCS ≥ 4) in that month were considered infected, as previously reported by Ruegg [25].

Cases of clinical mastitis were detected through forestripping during the milking routine, and high SCC cows were checked for clinical signs, according to Ruegg [26]. Detected animals were checked by the veterinary team, who took milk samples from the affected quarter for culture and antibiotic sensitivity [27] and recorded the case. The animals with mastitis were treated following a previously stipulated antibiotic protocol, modified by the sensitivity results if necessary.

Milk samples of affected quarters were taken in sterile vials, stored at −20 °C [28], and sent to the Northwest Regional Laboratory of DILAVE, Paysandú, Uruguay, where they were cultured on blood agar plates and incubated at 37 °C for 24–48 h to determine the microorganisms causing the disease [28].

The incidence of clinical mastitis was calculated as the number of new cases (cows presenting the disease for the first time) divided by the number of lactating animals in each treatment per month [25]. The cumulative incidence of clinical mastitis was calculated as the total number of cows exhibiting mastitis at any time in lactation divided by the number of lactating cows per treatment multiplied by 100.

The temperature (°C) and moisture (%) of the compost-bedded pack were measured weekly at a depth of 20 cm [29]. Bedding parameters were at acceptable levels during the entire period.

**Figure 1:**
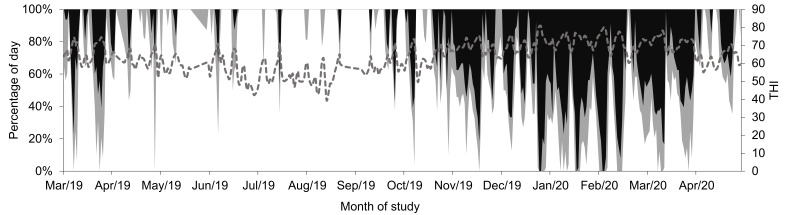
Percentage of the day with a temperature humidity index (THI) <68 (white area), 68 to 71 (grey area), and ≥72 (black area), and average THI (dotted grey line), during the months of study.

Daily rainfall (mm), environmental temperature, and relative humidity were recorded daily every half hour by the meteorological station of the experimental station. The temperature humidity index (THI) was calculated as (1.8 × ET + 32) − (0.55−0.0055 × RH) × (1.8 × ET − 26.8), where ET is environmental temperature, and RH is relative humidity [30]. The proportion of the day in each range of THI (<68, 68 to 71, ≥72) was calculated and is shown in Figure 1.

### 2.3. Statistical Analysis

Each season was analysed separately. Daily milk yield and SCS were analysed using a generalised linear mixed model (GLIMMIX procedure of SAS; SAS Institute Inc., Cary, NC, USA), with the treatment, week of the year, and interaction between the treatment and week as fixed effects. The block was considered a random effect. Post hoc comparisons were performed with a Tukey–Kramer test.

The UHS was analysed, classifying the data into two categories: “clean” (scores 1 and 2) and “dirty” (scores 3 and 4) [11]. For this analysis, a generalised linear mixed model for a binomial distribution variable was used (GLIMMIX procedure of SAS; SAS Institute Inc., Cary, NC, USA). The fixed effects were the treatment, date of observation of the UHS, and interaction between the treatment and the date of observation, and the block was considered as a random effect. The effect of rainfall (>30 mm) on UHS was studied by a chi-square test (PROC FREQ; SAS Institute Inc., Cary, NC) for each treatment, and the odds ratio (OR) and its 95% CI were reported.

To determine the correlation between the UHS and IMI and between the UHS and SCS, the closest measure of IMI and SCS after UHS observation (1 to 20 days; [11]) was used, and the Spearman test was carried out.

The relative prevalence of IMI was analysed using a generalised linear mixed model for a binomial distribution variable (GLIMMIX procedure of SAS; SAS Institute Inc., Cary, NC, USA), with fixed effects defined as the treatment, month, and interaction between the treatment and month, and the block with the animal nested as a random effect.

For the analysis of the monthly and cumulative incidence of clinical mastitis, contingency tables were made and analysed by Fisher’s exact test for binomial variables. The 95% confidence intervals were estimated by the Wilson Score method.

For all analyses, a value of *p* ≤ 0.05 was considered significant and a tendency when 0.05 < *p* ≤ 0.10.

The results of the bacterial cultures are presented as descriptive statistics according to the calving season for both treatments and include samples of cases of clinical mastitis (first and recurrent events; [25]).

## 3. Results

**Figure 2:**
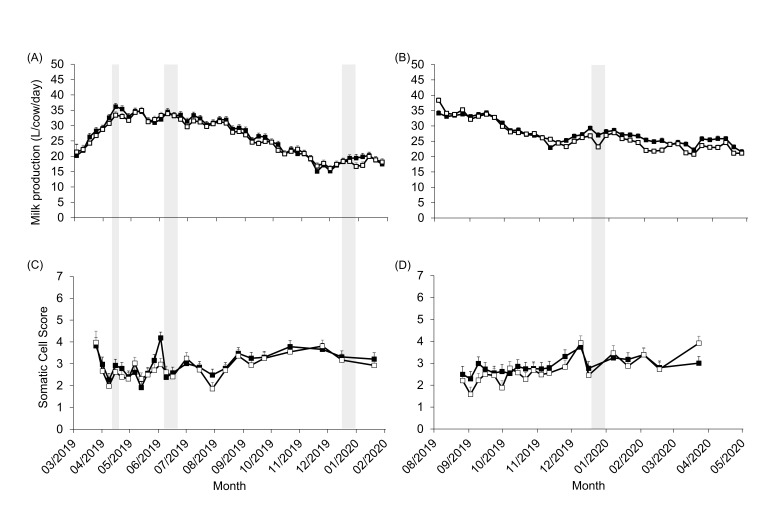
Milk production (L/cow/day; (**A**,**B**)) and somatic cell score (**C**,**D**) by week for compost barn (CB ◼) and outdoor soil-bedded (OD ☐) in autumn (**left**) and spring (**right**) calving cows. Grey bars indicate periods of complete confinement.

Milk production was not affected by the treatment but was affected by the week (*p* < 0.0001) and the interaction between the treatment and week (*p* < 0.0001) in both calving seasons. In autumn-calving cows, milk production gradually increased and reached a peak between the seventh and eighth week for CB cows and at the eleventh week for OD cows (Figure 2A). Milk production gradually decreased in both treatments, reaching its lowest values at the end of lactation, which coincided with the summer for autumn-calving cows. In spring-calving cows, the highest values of milk production coincided with the beginning of lactation. Milk production was lower in both treatments during the summer months. OD cows showed more weekly variation in these months, and production drops were observed during February and March (Figure 2B).

In both calving seasons, the SCS was not affected by the treatment but was affected by the week (*p* < 0.0001) and the interaction between the treatment and week (*p* < 0.05). The highest SCS was observed in the first week for both treatments in autumn-calving cows (Figure 2C) and in the first week of December for both treatments in spring-calving cows (Figure 2D).

The mean UHS were 2.0 and 2.6 for autumn-calving CB and OD cows, respectively, and 2.1 and 2.7 for spring-calving CB and OD cows, respectively. There was a treatment effect (*p* < 0.001) for the UHS classified as “clean” and “dirty” in both calving seasons, as OD cows were dirtier than CB cows. In the autumn-calving season, an average of 50.7% of OD cows were classified as dirty over the entire lactation, while in CB cows, this value was 17.4% (OR = 3.3, CI 95%: 2.2–4.9; *p* < 0.0001). In the spring-calving season, an average of 52.8% of the OD cows and 26.0% of the CB cows were classified as dirty (OR = 2.3, CI 95%: 1.5–3.7; *p* < 0.001) over the entire experimental period.

**Figure 3:**
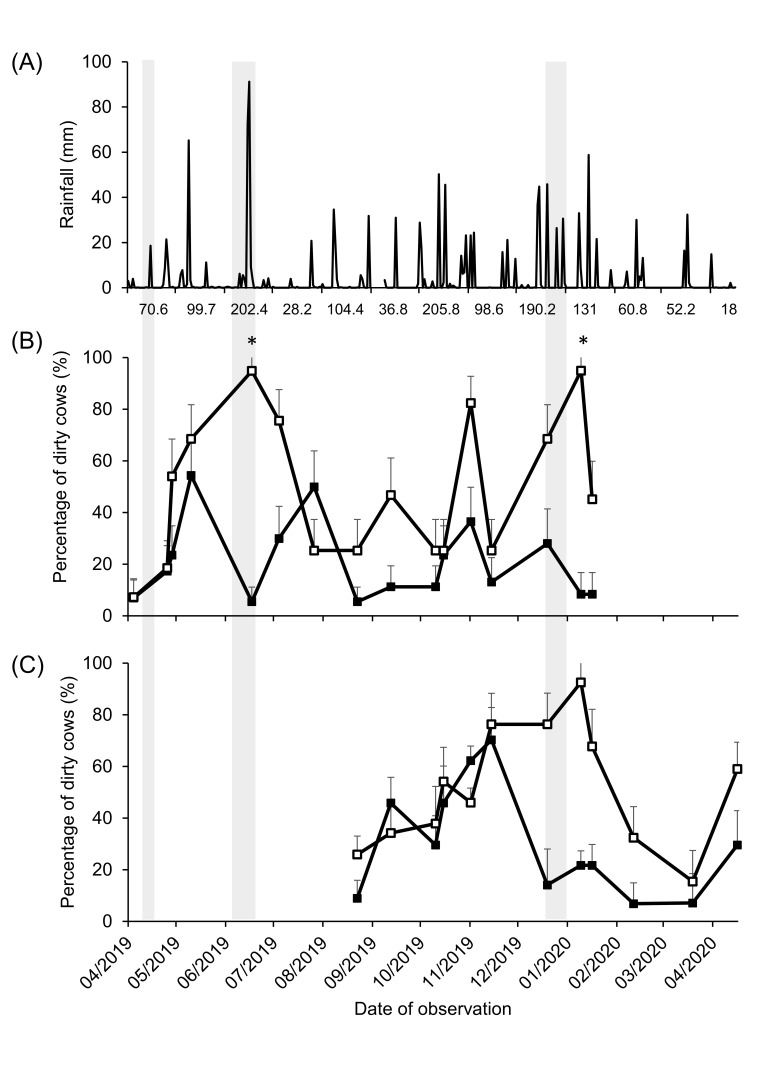
Daily and monthly accumulated rainfall (**A**), percentage of dirty cows (UHS score 3 and 4) per date of observation for compost barn (CB ◼) and outdoor soil-bedded (OD ☐), for autumn (**B**) and spring (**C**) calving cows during the experimental period. Grey bars indicate periods of complete confinement. Asterisks indicate significant differences between treatments in autumn-calving cows (*p* < 0.01).

Figure 3A shows the daily and monthly accumulated rainfall during the experimental period and the percentage of dirty cows in the autumn- and spring-calving seasons (Figure 3B,C). In autumn-calving cows, the interaction between the treatment and date of observation affected the percentage of dirty cows (*p* < 0.01; Figure 3B), as the percentage of dirty cows was higher in OD conditions in June and January compared to CB. In spring-calving cows, the interaction between the treatment and date of observation tended to be significant (*p* = 0.07), as OD cows were dirtier from November to January, while CB presented a higher percentage of dirty cows in November compared to subsequent months (Figure 3C).

Rainfall higher than 30 mm was associated with a higher percentage of dirty cows in the autumn-calving OD treatment with an OR = 1.85 (CI 95%: 1.1–3.1; *p* < 0.02). In CB cows, the percentage of dirty cows was not affected by rainfall. In spring-calving cows, rainfall did not affect the UHS in either treatment.

In autumn-calving OD cows, the UHS tended to be weakly correlated with IMI (r = 0.12; *p* = 0.07) and the UHS with SCS (r = 0.11; *p* = 0.09). These correlations were not significant in CB autumn-calving cows or both spring-calving treatments. For autumn-calving cows, the prevalence of IMI was not affected by the treatment, month, or interaction between the treatment and month. For spring-calving cows, there was no effect of treatment or treatment by month interaction on the prevalence of IMI (Table 1). There was a tendency for the month to have an effect (*p* = 0.06), as the prevalence of IMI tended to be higher in December than in November (45 ± 11.1% vs. 9 ± 6.1%). In either calving season, monthly and cumulative clinical mastitis incidence showed no significant differences between the treatments. The cumulative incidence of clinical mastitis is shown in Table 2.

Nineteen isolates were obtained for CB and eight for OD autumn-calving cows, and fifteen isolates for CB and eight for OD spring-calving cows. Over half of the samples showed no growth, regardless of the treatment and calving season. In OD, in both calving seasons, the isolated bacteria were *Escherichia coli* and *Streptococcus uberis*, while in CB, in addition to these bacteria, others such as *Klebsiella pneumoniae*, *Streptococcus dysgalactiae, Nocardia* spp., and coagulase-negative *Staphylococcus* were isolated.

## 4. Discussion

As far as we know, this is the first study to explore the association between contrasting housing facilities and udder hygiene score and mastitis indicators during half-time confinement in pasture-based systems. Our study presents information collected in a well-controlled field trial studying the impact of different environmental conditions throughout the entire lactation in autumn- and spring-calving cows. The percentage of dirty cows was higher in outdoor soil-bedded housing than in the compost barn in both calving seasons. In OD, rain was associated with a higher percentage of dirty cows, while in CB, the percentage of dirty cows was not affected by rain. Despite the higher percentage of dirty cows in OD, no difference in IMI between treatments was found.

The higher SCS in the first week recorded in autumn-calving cows is in accordance with Schepers et al. [31]. However, in spring-calving cows, the SCS was highest in December, and there was a tendency for a higher prevalence of IMI, which could be associated with heat stress. This was also reflected in milk production (Figure 2A), and these findings have been reported previously [17,18,32]. Both treatments showed an increase in SCS, although the CB contained facilities to mitigate heat stress during housing. Another possible explanation could be a dilution effect, given the inverse profile of the milk production and SCS curves [33].

The higher percentage of dirty cows in OD than in CB is consistent with the literature, which attributes this to muddy conditions of the environment [34,35] generated by trampling and animal excretions on the dirt floor, as well as by exposure to rainfall [16]. Similarly, O’Driscoll et al. [7] and Boyle et al. [20] found higher dirtiness scores in the outdoor housing on wood chips when compared to free-stalls, using dry cows and heifers, respectively. In contrast, Sjostrom et al. [9] found a higher UHS in lactating cows in a compost barn than in an outdoor straw pack. High moisture in the compost bedded pack and the frequent addition of bedding material to the outdoor straw pack could explain the difference [9]. In contrast to our study, animals in the aforementioned studies were in complete confinement and were only evaluated during the winter [7,9,20].

The interaction between the treatment and date of observation in the autumn-calving season showed a higher percentage of dirty cows in OD compared to CB during June and January. This could have been the result of higher rainfall, with an accumulated value of 202 mm in June, 190 mm in December, and 131 mm in January, and complete confinement due to low herbage mass in both periods (Figure 3A). In such conditions, trampling and an accumulation of excretions due to the increased time in confinement could have increased the formation of mud in OD, indicating that more intensive use of this housing system, plus the weather conditions, could cause worse results in terms of the UHS. The study by Sjostrom et al. [9] seems to suggest that the addition of a straw pack to the OD yard could have improved the UHS; however, this requires further investigation under Uruguayan conditions, as straw may contain very high numbers of environmental streptococci, in particular when humid [36].

Furthermore, when it rained more than 30 mm, autumn-calving OD cows were dirtier than on days without previous rain. Even with access to a pasture in both treatments, the greater exposition to environmental conditions in OD increased the percentage of dirty cows compared to CB. Weather conditions during the autumn-winter period, with a delay in the ground drying process due to low solar radiation [37] and ambient temperature (low evaporation), may have contributed to this effect since rainfall did not affect the UHS in spring-calving cows. Thus, the impact of OD housing on the UHS may be exacerbated depending on the season in which it is used.

In spring-calving cows, the UHS tended to be affected by the interaction between the treatment and date of observation, as OD cows had higher UHS from November to January, which could be the result of the same combination of rainfall and complete confinement. On the other hand, the spring-calving CB treatment presented a higher percentage of dirty cows in November, which may be associated with the moisture of the bedded pack at this moment, which was near the upper limit, as reported before [38,39,40].

A tendency for a correlation between the UHS and IMI, as well as between the UHS and SCS, was found only for OD autumn-calving cows and presented a small magnitude (r = 0.11 and r = 0.12, respectively). This magnitude of correlation is similar to the association between the UHS and SCS (R = 0.15) found by Reneau et al. [12] in dairy cows in complete confinement in free-stalls. This association—although weak—may be of relevance because most of the pathogens isolated in the present work were environmental bacteria, as has been observed internationally in recent years [41,42]. Nevertheless, no significant differences were detected between treatments in terms of IMI and the incidence of clinical mastitis. The low number of cows in this experiment may have been a limitation. Differences in UHS were associated with subclinical mastitis incidence [11], although other studies did not find an association between the dirtiness score and the incidence of clinical mastitis [43] or a newly raised cell count [8]. There may be other factors that affect the correlation between UHS and mastitis incidence and prevalence among different farms. Large-scale data from the USDA [44] revealed that, despite the fact that cows in dry-lots had a higher UHS compared to free-stalls, the incidence of mastitis was higher in the free-stalls. Different bedding types have different bacterial loads [45,46,47], but few studies have found a strong correlation between bedding materials and mastitis. Sjostrom et al. [9] found a higher incidence of clinical mastitis in cows in a compost barn compared to an outdoor straw-pack during the winter without access to grazing. On the other hand, in a survey of commercial dairy farms in Brazil with outdoor systems that migrated to total confinement in compost barn systems, no differences were found in terms of the SCC or treatment costs [48]. As mentioned, the management conditions in the cited studies differed from the present study. We did not find studies that evaluated the UHS and the incidence of mastitis in pasture-based systems, comparing confinement in outdoor paddocks with a compost barn during the entire lactation.

## 5. Conclusions

The cows confined in OD presented a higher percentage of dirty cows compared to CB in both calving seasons. Rainfall of more than 30 mm was associated with a greater percentage of dirty cows in OD cows calving in autumn but not in CB cows. There was a tendency for a weak correlation between the UHS and IMI and between the UHS and SCS only for autumn-calving cows in the OD system. The negative effect on UHS in OD cows was exacerbated by heavy rainfall and complete confinement. Contrary to our hypothesis, we did not find strong evidence for its association with mastitis indicators in this experiment, reaffirming the complex nature of this disease.

## Figures and Tables

**Table 1:** Prevalence of intramammary infection (IMI; mean ± standard error) for cows in the compost barn (CB) or outdoor soil-bedded (OD) in the autumn and spring-calving seasons.

	Treatments		*p*-Value		
	CB	OD	Treat	Month	Treat × Month
Autumn	34 ± 8.9	28 ± 8.8	ns	ns	ns
Spring	20 ± 8.2	24 ± 9.5	ns	0.06	ns

**Table 2:** Cumulative clinical mastitis [mean (95% confidence interval)] for cows in the compost barn (CB) or outdoor soil-bedded (OD) in the autumn and spring-calving seasons.

	Treatments		*p*-Value
	CB	OD	Treat
Autumn	31 (14–56)	53 (30–75)	ns
Spring	54 (29–77)	50 (27–73)	ns

## Data Availability

Not applicable.
